# American Association of Obstetricians and Gynecologists

**Published:** 1892-11

**Authors:** 


AMERICAN ASSOCIATION OF OBSTETRICIANS AND GYNECOLOGISTS, ST. LOUIS, MISSOURI, SEPTEMBER 20-23, 1892.

DR. GEORGE H. ROHE, of Catonsville, Maryland, read a pape upon "The Relations of Pelvic Diseases to Psychical Disturbances in Woman."

The author pointed out the frequency with which bodily conditions influenced mental states. Thus a torpid condition of the intestines, Bright's disease, putrefactive processes in the intestinal canal, etc., might give rise to melancholia and other disorders of the mental functions. It is not irrational to suppose likewise that diseases of the female sexual apparatus would have a not inconsiderable influence in the production or perpetuation of mental disorders. As a contribution to the knowledge of the subject the following report was submitted :

In a hospital containing 200 insane women, thirty-five were subjected to vaginal examination and twenty-six found with evidences of pelvic diseases. In eighteen of these the uterine appendages were removed with the following results :

Sixteen recovered from the operation and two died. Of the sixteen recovered, three have been discharged from the hospital completely restored, both physically and mentally. In ten considerable improvement followed the operation in both physical and mental conditions, and in three the operation was of too recent a date to allow any definite expression of opinion.

The mental disorders present in the eighteen cases was melancholia in six cases, simple mania in one, puerperal mania in four, hysterical mania in one, periodic mania in two, hystero-epilepsy with mania in one, and epilepsy with mania in three.

The author basing his opinion upon his experience, concludes as follows :

"The facts recorded demonstrate first ; that there is a fruitful field for gynecological work among insane women ; second, that this work is as practicable and can be pursued with as much success in an insane hospital as elsewhere ; and third, that the results obtained not only encourage us to continue in the work, but require us, in the name of science and humanity to give to an insane woman the same chance of relief from disease of the ovaries and uterus that a sane woman has."

Dr. Rufus B. Hall, of Cincinnati, Ohio, read a paper entitled "Six Consecutive cases of Extra-Uterine Pregnancy and the Lesson they Teach."

The author said he would try to illustrate and emphasize a few facts in connection with the subject of extra-uterine pregnancy, which are of great practical importance to the general practitioner and the specialist alike. He illustrated from clinical facts the difficulty attending a correct diagnosis as to intra and extra-peritoneal rupture of the sac in extra-uterine pregnancy in the early months of gestation, and the danger to the patient in attempting the same, thus encouraging delay in making the necessary operation in those cases where the rupture has occurred. In the six cases reported as the basis of his paper five had ruptured before the operation was made. In all the ruptures had occurred from three to four weeks before the operation, and in every instance the sac showed conclusively that the rupture was free into the peritoneal cavity from the first and not into the folds of the broad ligament. The author dwelt upon the fact that in two of the cases, the first and the fourth in the series, the blood clot had become so firm by the absorption of the fluid portion of the blood, and from the adhesions of intestine and omentum above so as to depress the pelvic floor, making it appear from the physical examination that the hemorrhage was really in the folds of the broad ligament. That all recovered when we consider the clinical history in the individual cases must be considered in the nature of a happy surprise. The lesson conveyed in the report was that there are no certain means of knowing before the operation whether or not the rupture has taken place into the peritoneal cavity or the broad ligament; especially is this true if the rupture occurs in the first few weeks of gestation. Therefore, if we treat all cases of rupture as if they were really ruptures into the peritoneal cavity it would be the correct practice. The author of the paper believes if a case comes under observation before the fourth month of gestation it is the duty of the physician not to wait until he is certain that rupture has taken place into the peritoneal cavity before advising an operation, but to give the patient the best chance for her life—and that is an abdominal section—and that without delay.

DR. CHARLES A. L. REED of Cincinnati, presented a "Report of Twenty-five Cases of Complete Vaginal Extirpation of the Womb for Cancer," with only two primary deaths—one from shock and one from iodoform poisoning. Of the twenty-five that were operated upon but fourteen were of more than two years standing, and hence were all that could be discussed with reference to their ultimate results. These fourteen were divisible into two classes of seven each, viz. : those in which the disease had existed for more than six months before the operation, and those in which it had existed for less than six months before the operation. Of the first class, that is, those of more than six months' (an average of ten months) previous duration, all were dead ; of the second class, that is, those of less than six months' (an average of four months) previous duration, only one has since died. One of the recoveries is of more than five years' duration. The conclusion from these figures is that cases of cancer of the uterus ought to be remanded for operation as soon as diagnosed. Dr. Reed looks upon total extirpation as the only operation to be advised or practiced in these cases, the primary mortality from which, in experienced hands, varies from five to eight per cent.

